# Differential financing of public health facilities

**DOI:** 10.1186/1753-6561-6-S5-O10

**Published:** 2012-09-28

**Authors:** Nehal Jain, Bhavesh Jain, Prakhya Bhat, MR Mohanraju

**Affiliations:** 1 Centre for Budget and Policy Studies, Karnataka, India; 2 Karnataka State Health System Resource Centre, Karnataka, India

## Introduction

The health system in India suffers from low level of public spending and high out of pocket payment. Public health facilities are funded on a fixed and uniform basis irrespective of their needs and performance. The National Rural Health Mission has introduced an innovative approach of flexible financing to public health facilities under Untied Funds (UF), Annual Maintenance Grant (AMG) and Aarogya Raksha Samithi Corpus Funds (ARS). Funds are currently allocated @ of INR 10000 at village level, INR 20000 at subcentre level, INR 175000 at Primary Health Centre, INR 250000 at community health centre and INR 500000 at district hospital level. Though funds are allocated to health facilities with adequate guidelines and flexibility, utilization of these funds is poor. Given the limited resource available for healthcare in the country, there is a need to rationalize the distribution of funds and allocate them efficiently. We undertook a study to propose a differential financing model for public health facilities.

## Methods

We conducted a cross sectional study of 46 primary and secondary level facilities in two districts of Karnataka in the year 2011-12. Retrospective data on resources, finance and performance of 2008-09, 2009-10 and 2010-11 were collected. In addition, interviews were conducted with members of ARS committee responsible for taking decisions on utilization of funds.

## Results and discussion

The study found that utilization of funds in health facilities are influenced by several factors. These included per-capita allocation, distance from district headquarter, availability of equipment and infrastructure.

We observed that there was no definite trend of impact of availability of human resources, number of beds, staff residing at the facility compound, training, cooperation among members and availability of guidelines on utilisation of funds at health facilities.

The study strongly advocates the need for a more rational and systematic planning for utilization of funds allocated to health facilities taking into consideration other health system constraints. The study proposes a formula for allocating funds on differential financing method: at the village level committee at INR 10 per-capita, at the sub-centre level at INR 4 per-capita, for primary health centre at INR 6 per-capita, at the level of community health centre at INR 1.04 per-capita and at INR 4167 per functional bed and the current norm of INR 500000 per district hospital. Such a model would result in efficient utilization of funds, leading to better of quality of delivery of services.

## Competing interests

None declared

## Funding statement

The study was funded by the Karnataka State Health System Resource Centre, Bangalore and the Centre for Budget and Policy Studies, Bangalore.

